# Risk factors of synchronous multifocal necrotizing fasciitis: a case control study in comparison with monofocal necrotizing fasciitis in Taiwan

**DOI:** 10.1186/s12879-019-4103-3

**Published:** 2019-06-11

**Authors:** Ching-Yu Lee, Tsan-Wen Huang, Meng-Huang Wu, Tsung-Jen Huang, Yan-Rong Li, Evelyn Jou-Chen Huang, Yao-Hung Tsai

**Affiliations:** 1 Department of Orthopedics, Taipei Medical University Hospital, No. 252, Wuxing St., Taipei, 11031 Taiwan; 2 Department of Orthopaedics, School of Medicine, College of Medicine, Taipei Medical University, No. 250, Wuxing St., Taipei, 11031 Taiwan; 3 Graduate Institute of Clinical Medical Sciences, College of Medicine, Chang Gung University, No. 259, WenHua 1st Rd., Guishan Dist., Taoyuan City, 33302 Taiwan; 4 Department of Orthopedic Surgery, Chiayi Chang Gung Memorial Hospital, No. 6, West Sec., Jiapu Road, Puzi City, Chiayi 613 Taiwan; 5 Department of Ophthalmology, Taipei Medical University Hospital, No. 252, Wuxing St., Taipei, 11031 Taiwan; 6 Department of Ophthalmology, School of Medicine, College of Medicine, Taipei Medical University, No. 250, Wuxing St., Taipei, 11031 Taiwan; 7 Department of Internal Medicine, Division of Endocrinology and Metabolism, Chang Gung Memorial Hospital, No. 5, Fuxing St., Guishan Dist., Taoyuan City, 33305 Taiwan

**Keywords:** Synchronous multifocal necrotizing fasciitis

## Abstract

**Background:**

Monofocal necrotizing fasciitis (MONF) involves a single site in a rapidly progressing infection and necrosis of the fascia and surrounding soft tissue. Synchronous multifocal necrotizing fasciitis (SMNF), the simultaneous development of NF in multiple noncontiguous sites, is rarely reported. This study aimed to compare the clinical characteristics and outcomes between patients with SMNF and MONF, and to determine the risk factors of SMNF.

**Methods:**

Our retrospective case-control study compared the clinical characteristics and outcomes, between January 2006 and January 2013, of patients with SMNF and of patients with MONF of the extremities.

**Results:**

We enrolled 144 patients with NF of the extremities: 19 with SMNF and 125 with MONF. The duration of symptoms before admission was significantly shorter for the former than for the latter (1.7 days vs. 3.3 days, *p* = 0.001); the prevalence of shock at the initial visit significantly higher (73.7% vs. 36%, *p* = 0.002); and the total-case postoperative mortality rate significantly higher (68.4% vs. 14.4%, *p* <  0.001). In further analysis of the total-case mortality, 9 in 13 SMNF deaths (69.2%) within 7 days after fasciotomy were in the majority while 13 with 28-day mortality (72.2%) was the majority of MONF deaths (*p* <  0.001). SMNF was significantly more likely to involve bacteremia (89.5% vs. 36%, *p* <  0.001). Independent risk factors for SMNF were liver cirrhosis (LC) (odds ratio [OR] 6.0, *p* = 0.001) and end-stage renal disease (ESRD) (OR 7.1, *p* = 0.035). Gram-negative bacteria were most common in SMNF, and Gram-positive bacteria in MONF (83.3% vs. 53.3%, *p* = 0.005). Vibrio species were the most common single microbial cause (35.4%) of all NF patients and were the overwhelming cause (73.7%) of SMNF. Staphylococcus aureus and group A β-hemolytic streptococcus (45.6%) were the other predominant causes of MONF while both (10.5%) rarely caused multifocal NF.

**Conclusions:**

*S*MNF was more fulminant than was MONF. SMNF was attributable primarily to marine Gram-negative bacteria. Physicians should be aware of SMNF because of its extremely high mortality rate.

## Background

Necrotizing fasciitis (NF) is a life-threatening deep soft tissue infection characterized by rapidly progressive necrosis of the fascia and the adjacent soft tissue. It is well known that monofocal necrotizing fasciitis (MONF) is typical of NF: the extremities are the most common site of infection [1]. The overall mortality rate has been estimated to be 21.9% [2, 3]. Synchronous multifocal necrotizing fasciitis (SMNF), the simultaneous development of NF in multiple noncontiguous sites, is rarely reported in the literature [4, 5]. We previously reported that SMNF has a mean mortality rate of 67%, much higher than that reported for MONF [6]. However, there are no reports that compare clinical outcomes of patients with SMNF to those of patients with MONF. Thus, we wanted to determine and clarify the differences in clinical characteristics and outcomes between patients with SMNF and those with MONF.

## Methods

We reviewed the records of patients discharged from Chang Gung Memorial Hospital, Chiayi, between January 2006 and January 2013 with the International Classification of Diseases, Ninth Revision (ICD-9) NF diagnosis code of 72,886. The current study included 18 patients with SMNF from the previous case-series report [6] and was a further analysis of a case-control study to compare clinical characteristics and outcomes of patients with SMNF to those of patients with MONF.

**Fig. 1:**
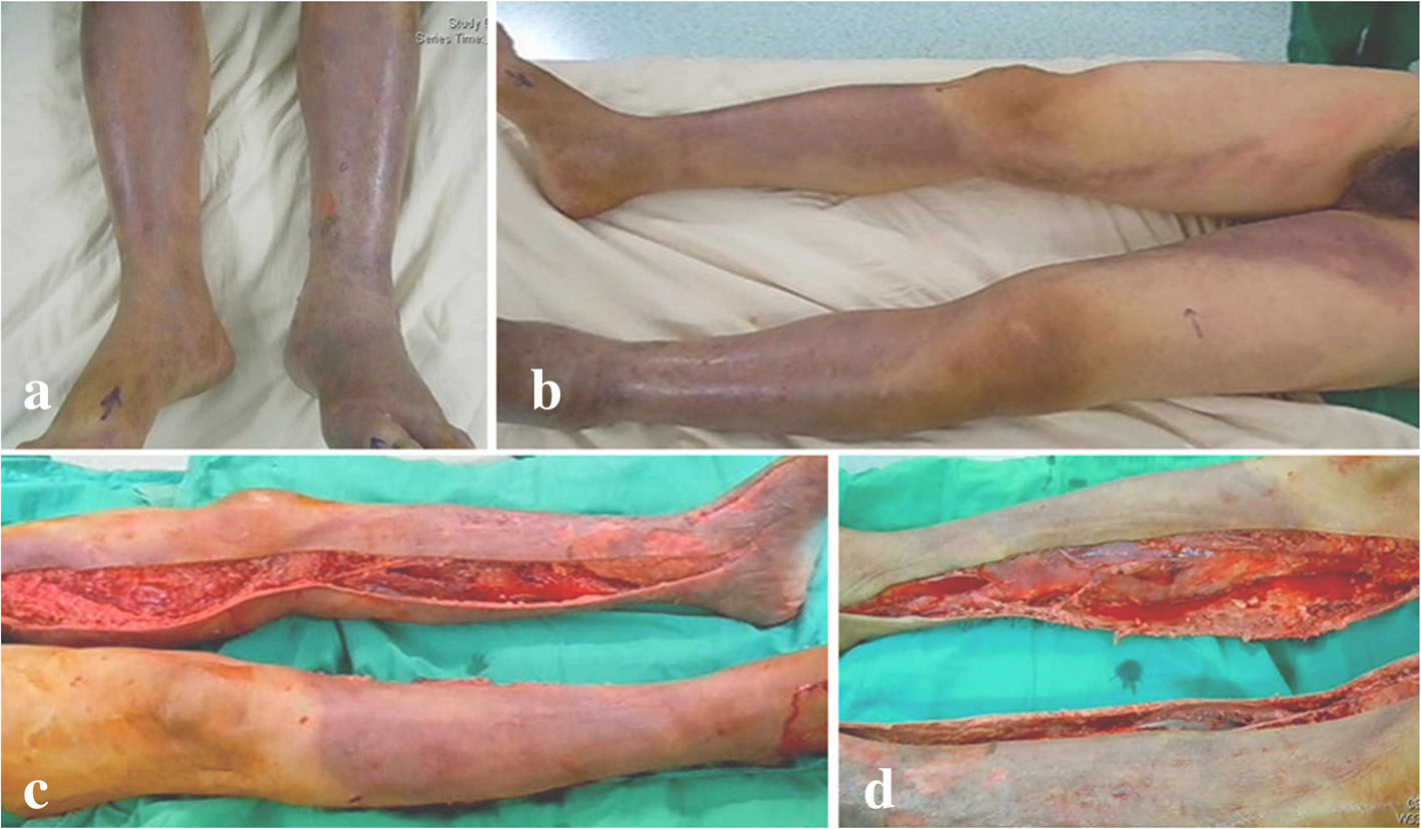
Synchronous multifocal *Vibrio* spp. necrotizing fasciitis on both lower limbs in a 60-year-old fisherman with multiple comorbidities: liver cirrhosis and end-stage renal failure. **a** An acute onset of intractable pain, swelling, purpura, and hemorrhagic bullae occurred in both feet on the second day after saltwater exposure. **b** The swollen and purpuric lesions with out of proportion pain were rapidly worsening and extending to both thighs. **c** and **d**. Loss of resistance of normal adherent superficial fascia to gentle finger dissection was found. Immediate fasciotomies on both lower limbs revealed foul-smelling fluid. The patient died of uncontrolled sepsis 4 days after the onset of synchronous multifocal necrotizing fasciitis

NF was defined by surgical findings: the presence of grayish necrotic soft tissue, loss of resistance of normally adherent fascia to gentle finger dissection, and the presence of pus with the foul odor of dishwater (Fig. 1). Histopathological tissue specimens were obtained to confirm the diagnoses. SMNF was defined as a multifocal presence of necrotizing fasciitis in different extremities on the initial visit. MONF was defined as the occurrence of necrotizing fasciitis in a single extremity. Exclusion criteria were: (1) necrotizing deep soft tissue infections involving the cervical region, the trunk, or the perineum, (2) negative bacterial cultures from the infected specimen, (3) comorbid infections (e.g., osteomyelitis, surgical wound infection), and (4) a preexisting chronic wound (e.g., diabetic foot ulcer and decubitus ulcer).

The treatment protocol included antibacterial therapy and surgical debridement. All patients underwent emergency surgery after they had been admitted if NF was suspected. Blood cultures were done in the Emergency Department before antibiotic therapy was started. During surgical debridement, multiple tissue samples were obtained from the necrosis of fascia and subcutaneous tissue for cultures of aerobic, anaerobic, fungal, and mycobacterial organisms. Surgical debridement was done every other day if progressive necrotic changes were combined with a deteriorating clinical presentation. Initial broad-spectrum antibiotics were administered and adjusted based on the results of blood cultures and tissue tests. Soft tissue reconstruction was done until the infected necrotic tissue was controlled and stabilized. All patients included in this study were followed-up for a minimum of 2 year.

### Data analysis

To compare the clinical manifestations and outcomes between patients with SMNF and patients with MONF, we reviewed their clinical characteristics, comorbidities, laboratory data at the time of admission, bacteriological results, and final outcomes of all patients in our hospital’s electronic database. The comorbidity of shock was defined as systolic blood pressure < 90 mmHg or 20 mmHg below the patient’s usual pressure or mean arterial pressure < 65 mmHg, leukocytosis as white blood cell count > 12,000/μL; of bandemia as the percentage of immature neutrophils > 5%; and of thrombocytopenia as a platelet count < 150,000 mm^3^. To assess clinical outcomes after antibacterial therapy and surgical debridement, the total-case postoperative mortality was defined as the number of postoperative death within 6 months due to progressive sepsis or medical complications. For further analysis of the total-case mortality, the total-case mortality was divided into 7-day, 28-day and 6-month mortality. The 7-day mortality was defined as death within 7 days after fasciotomy, the 28-day mortality was defined as death from the 8th day to 28th day after fasciotomy, the 6-month mortality was defined as death from the 29th day to 180th day after fasciotomy.

### Statistical analysis

Univariate analysis was used to determine factors associated with SMNF and MONF. An independent Student’s *t* test was used for numerical data. A χ^2^ test or a Fisher’s exact test was used for categorical data. Descriptive data are presented as the mean ± standard deviation for quantitative variables and as frequency for categorical variables. Significance was set at *p* <  0.05. Multivariate analysis was used to determine independent risk factors associated with underlying comorbidities. All factors significant in univariate analysis were included in a multivariate logistic regression analysis. SPSS 12.0 for Windows was used for all statistical analyses.

## Results

### Patient characteristics compared

We identified 351 patients at our hospital who had NF between January 2006 and January 2013. We excluded 207 patients: 80 with NF involving the trunk, 79 with a diabetic foot ulcer or a decubitus ulcer, 35 with comorbid chronic osteomyelitis or a surgical wound infection, and 13 with culture-negative NF. One hundred forty-four patients with NF of the extremities were enrolled: 19 with SMNF and 125 with MONF (Table 1). Nineteen patients with SMNF included a newly added patient and 18 from the previous study [6]. The duration of symptoms before admission was significantly shorter for SMNF patients than for MONF patients (1.7 vs. 3.3 days, *p* <  0.001). The prevalence of shock at the initial visit was significantly more frequent in SMNF patients than in MONF patients (74% vs. 36%, *p* = 0.002). End-stage renal disease (ESRD) and liver cirrhosis (LC) were significantly more frequent in SMNF patients than in MONF patients (*p* = 0.03 and *p* = 0.001, respectively). the total-case postoperative mortality rate was significantly higher in SMNF patients than in MONF patients (14% vs. 68%, *p* <  0.001). In further analysis of the total-case mortality, In further analysis of the total-case mortality, 9 in 13 SMNF deaths (69.2%) within 7 days after fasciotomy were in the majority while 13 with 28-day mortality (72.2%) was the majority of MONF deaths (*p* <  0.001).

**Table 1:** Comparison of patient characteristics

	SMNF (n = 19)	MONF (*n* = 125)	*P*
Age, mean (SD), year	60 (12)	65 (15)	0.195
Males, No. (%)	15 (78.9)	93 (74.4)	0.783
Duration of symptoms, mean (SD), day	1.7 (1.2)	3.3 (2.6)	< 0.001*
Shock during initial visit, No. (%)	14 (73.7)	45 (36)	0.002*
Comorbidity, No. (%)			
Diabetes mellitus	4 (21.1)	48 (38.4)	0.2
End-stage renal disease	3 (15.8)	3 (2.4)	0.03*
Alcoholism	6 (31.6)	28 (22.4)	0.38
Liver cirrhosis	9 (47.3)	16 (12.8)	< 0.001*
Hepatitis B or Hepatitis C virus	6 (31.6)	44 (35.2)	0.757
Hepatocellular carcinoma	2 (10.5)	3 (2.4)	0.13
Total-case mortality, No. (%)	13 (68.4)	18 (14.4)	< 0.001*
7-day mortality	9 (69.2)	1 (5.6)	< 0.001*
28-day mortality	2 (15.4)	13 (72.2)	
6-month mortality	2 (15.6)	4 (22.2)	

*SMNF* synchronous multifocal necrotizing fasciitis; *MONF* monofocal necrotizing fasciitis, *SD* standard deviation; *p < 0.05

### Multivariate analysis of independent risk factors for SMNF

Independent risk factors for SMNF were LC (odds ratio [OR] = 6.0, 95% CI = 2.0–17.5, *p* = 0.001) and ESRD (OR = 7.1, 95% CI = 1.1–44, *p* = 0.035) (Table 2).

**Table 2:** Multivariate analysis of independent risk factors

Risk factor for SMNF	*P*	OR (95% CI)
Liver cirrhosis	0.001*	6.0 (2.0–17.5)
End-stage renal disease	0.035*	7.1 (1.1–44.0)

*SMNF* synchronous multifocal necrotizing fasciitis, *OR* odds ratio;

*CI* confidence interval; *p < 0.05

### Laboratory data compared

The prevalence of leukocytosis was significantly (*p* = 0.016) lower in SMNF patients than in MONF patients (37% vs. 66%) (Table 3). SMNF patients had a greater band count than did MONF patients (14.8% vs. 5.2%). The prevalence of thrombocytopenia was higher in SMNF patients than in MONF patients (68% vs. 43%). Elevated C-reactive protein levels were not significantly (*p* = 0.281) different in SMNF and MONF patients (111 mg/L vs. 146 mg/L). The prevalence of bacteremia was significantly (*p* = 0.001) higher in SMNF patients than in MONF patients (89% vs. 36%).

**Table 3:** Comparison of laboratory data

Laboratory data	SMNF (n = 19)	MONF (n = 125)	*P*
Leukocytosis (≥ 12,000/μL), No. (%)	7 (36.8)	82 (65.6)	0.016*
Bandemia (> 5%), No. (%)	14 (73.7)	48 (38.4)	0.004*
Thrombocytopenia (< 150,000 mm^3^), No. (%)	13 (68.4)	54 (43.2)	0.04*
C-reactive protein, mean (SD), mg/dL	111 (98)	146 (127)	0.281
Bacteremia, No. (%)	17 (89.5)	45 (36)	< 0.001*

*SMNF* synchronous multifocal necrotizing fasciitis, *MONF* monofocal necrotizing fasciitis, *SD* standard deviation; *p < 0.05

### Bacterial cultures compared

A single bacterial species was isolated in almost every patient with SMNF (95%) or MONF (96%) (*p* = 0.579). Gram-negative bacteria were significantly (*p* = 0.005) more frequently isolated from SMNF patients (83%) than from MONF patients (47%) (Table 4).

**Table 4:** Comparison of bacterial cultures from necrotic tissue

	SMNF (n = 19)	MONF (n = 125)	*P*
Monomicrobial isolation, No. (%)	18 (94.7)	120 (96)	0.579
Gram-positive bacteria	3 (16.6)	64 (53.3)	0.005*
Gram-negative bacteria	15 (83.3)	56 (46.6)	
Polymicrobial isolation, No. (%)	1 (5.3)	5 (4)	
A total of bacterial species			
*Staphylococcus aureus*	1	31	
Methicillin-sensitive *Staphylococcus aureus*	0	16	
Methicillin-resistant *Staphylococcus aureus*	1	15	
Coagulase-negative staphylococcus	1	8	
*Streptococcus viridans*	0	3	
Group A β-hemolytic streptococcus	1	26	
*Enterococcus*	0	3	
*Vibrio* spp.	14	37	
*Vibrio vulnificus*	13	36	
*Vibrio cholerae*	1	1	
*Aeromonas* spp.	2	7	
*Klebsiella* spp.	0	5	
*Pseudomonas aeruginosa*	0	2	
*Morganella morganii*	0	3	
*Proteus*	0	2	
*Escherichia coli*	0	1	
*Klebsiella pneumoniae*	1	0	
*Chryseobacterium* spp.	0	1	
*Shewanella putrefaciens*	0	1	
*Eikenella corrodens*	0	1	

*SMNF*, synchronous multifocal necrotizing fasciitis, *MONF* monofocal necrotizing fasciitis; *p < 0.05

## Discussion

This is the first study to compare the clinical presentation, microbiological characteristics, and outcomes of SMNF patients with those of MONF patients. Earlier studies have reported that from the onset of NF symptoms to seeking medical care was approximately 1–4 days [7, 8], but we found a mean of 1.7 days for SMNF patients, and a mean of 3.3 days for MONF patients. Additionally, SMNF patients had a higher incidence of septic shock at the initial visit. Therefore, more SMNF patients developed a hyperacute course with necrosis that rapidly progressed to systemic features of sepsis.

The current study indicated that ESRD and LC were independent risk factors for SMNF. The patients with ESRD or LC frequently have chronic cutaneous lesions on multiple extremities, e.g., vasculitis [9], pruritus acquired perforating dermatosis [10, 11], chronic ulceration [12], bullae disorders [10, 13], and bleeding purpura [13], which may serve as multiple entry ports on upper or lower extremities for bacterial invasion. In addition, our hospital is on the northern border of the Tropic of Cancer on the southwestern coast of Taiwan, where the local population makes a living by working in the commercial fishing industry or in aquaculture. Oyster farming comprises a large proportion of aquaculture in southwestern Taiwan. *Vibrio* species naturally occur in warm coastal areas and are plentiful in shellfish, especially in oysters [14, 15]. Furthermore, the highest prevalence rates of chronic viral hepatitis, alcoholic hepatitis, liver cirrhosis, and ESRD are in southwestern Taiwan [16]. Chronic liver diseases and ESRD increase the virulence of *Vibrio* spp. and are well known as risk factors for *Vibrio* soft tissue infections [17–21]. Therefore, patients with ESRD or LC may be susceptible to multiple-site Vibrio infection if both hands or legs are exposed to aquaculture water or handling of shellfish or oyster.

The etiology of multifocal necrotizing fasciitis may be not only simultaneous inoculation into multiple extremities but also bacteremia spreading to other extremities. Immunocompromise increases the risk of bacteremia and the current study found that SMNF patients (89%) had a much higher prevalence of bacteremia than MONF patients (36%). Several case reports indicated that immunocompromised patients can so rapidly develop multifocal NF due to metastatic septic embolization before they arrive at hospital [22, 23], that synchronous multifocal involvement in NF will be considered when the patients present to the Emergency Department. We hypothesize that the estuary environment, pathogenic microorganisms, and host factors are the three main factors that contribute to SMNF infections.

Microbiologically, NF is categorized into three types: type I is a polymicrobial infection with aerobic and anaerobic bacteria; type II is a Gram-positive bacterial infection, including group A *Streptococcus* spp. and *S. aureus*; type III is a Gram-negative bacterial infection [24]. We found that type III infections (83%) were more frequently in SMNF patients, whereas type II infections (53%) were more common in MONF patients. One systematic review of necrotizing fasciitis involving extremities reported that *S. aureus* was the most commonly isolated, and then group A streptococcus, and, finally, other infrequently isolated aerobic and anaerobic pathogens [2]. Vibrio species was the most common single microbial cause (35%) of all our NF patients and was the overwhelming cause (74%) of SMNF. Staphylococcus aureus and group A β-hemolytic streptococcus (46%) were the other predominant causes of MONF while both (11%) rarely caused multifocal NF. Thus, empiric antibiotics therapy for NF in coastal regions must always include regimens that treat Vibrio species, especially for multifocal NF.

Our analysis of our patients’ laboratory data upon admission showed significant differences between patients with SMNF and patients with MONF. Patients with SMNF had a higher prevalence of bandemia, thrombocytopenia, and bacteremia. Bandemia implies a release of immature neutrophils into the blood, indicative of sepsis. Thrombocytopenia frequently occurs in patients with severe septicemia, and it has proved to be a strong predictor of organ failure and death [25, 26]. Therefore, the compared laboratory results might indicate that SMNF rapidly exhausts the immune system and leads to overwhelming sepsis.

We found that the total-case postoperative mortality rate of SMNF (68%) was significantly higher than that of MONF (14%). In further analysis of total-case mortality, 69% of all deaths with SMNF occurred within 7 days after fasciotomy while 72% of deaths with MONF occurred between the second and fourth week. One study reported that the estimated SMNF mortality rate was 62.4% in South Korea, and that a fasciotomy would not improve the survival rate in patients with more than two necrotic areas [27]. They also said that SMNF was more likely associated with a poor prognosis, and that it caused a high incidence of amputation in survivors. A large area of necrotic destruction of the epidermis, subcutaneous tissue, fascia, and underlying muscles will increase the rate of morbidities like amputation. Therefore, patients with SMNF would present with higher and more immediate mortality and a poorer prognosis than would patients with MONF.

This study has some limitations. First, it was a retrospective case-control study. To minimize selection bias, we focused on patients with tissue-culture-positive NF of the extremities and excluded those with central NF and pre-existing diabetic and decubitus ulcers, which might have different etiologies and treatments from NF of the extremities. Central NF, which is associated with the neck and perineum, often arise from internal organ infections. Cervical NF has an odontogenic source of infection (or a peritonsillar abscess) [28]; perineal NF, Fournier’s gangrene has a urogenital origin of infection (or an anorectal abscess) [29]. Infectious complications of diabetic or decubitus ulcers frequently include pyomyositis or osteomyelitis. It is crucial to recognize the original infectious disease when treating central NF and diabetic or decubitus ulcers with a secondary infection. Second, despite our study having the largest sample of patients with SMNF in the literature (*n* = 19), the sample was still too small to allow us to make strong generalizations based on our data. Third, there was a lack of investigation of the efficacy of the adjunctive therapies, such as hyperbaric oxygen (HBO) and intravenous immunoglobulin (IVIG). Adjuvant HBO therapy improves the bactericidal function of neutrophils, fibroblast proliferation and collagen synthesis, which are important in infection control and wound healing [30]. In our institute, HBO was included in the treatment protocol for necrotizing fasciitis, and NF patients would undergo HBO therapy after surgical intervention [31]. However, our HBO chamber only allows for patients with stable vital signs. Most SMNF patients did not undergo HBO therapy because those patients with unstable hemodynamics and mechanical ventilation died a few days after emergent surgical fasciotomy. IVIG contains antibodies that neutralize streptococcal exotoxin superantigens and is considered as adjuvant therapy in NF patients with streptococcal toxic shock syndromes [32]. Group A streptococcus was an infrequent pathogen of SMNF in this study; however, IVIG may be not sufficiently effective in the treatment of SMNF.

## Conclusions

Almost all SMNF patients presented with more fulminant sepsis than did MONF patients at their initial visit. SMNF patients also had a more rapid onset of symptoms and a higher incidence of septic shock. ESRD and LC were independent risk factors for SMNF. Marine *Vibrio* species was predominantly isolated in SMNF, but *S. aureus* and GABHS were the major pathogens isolated in MONF. The incidence of SMNF was infrequent and accounted for 13% of all patients with NF of the extremities; however, the mortality rate of SMNF was 68%, significantly higher than the 14% mortality rate of MONF. Because SMNF is often lethal, patients with ESRD or LC are advised to avoid direct contact with seawater or fish. They should wear waterproof gloves, chest waders and boots when being exposed to seawater or processing fish [33]. Early recognition of SMNF without a misdiagnosis, sufficient resuscitation, immediate surgical treatment, and prompt antibiotic therapy are crucial for treating SMNF.

## Abbreviation

ESRD: End-stage renal disease
GABHS: Group A β-hemolytic streptococcus
ICD-9: International classification of diseases, ninth revision
LC: Liver cirrhosis
MONF: Monofocal necrotizing fasciitis
NF: Necrotizing fasciitis
SMNF: Synchronous multifocal necrotizing fasciitis
